# Immunotoxicological, biochemical, and histopathological studies on Roundup and Stomp herbicides in Nile catfish (*Clarias gariepinus*)

**DOI:** 10.14202/vetworld.2016.638-647

**Published:** 2016-06-23

**Authors:** Gihan G. Moustafa, F. E. Shaaban, A. H. Abo Hadeed, Walaa M. Elhady

**Affiliations:** Department of Forensic Medicine and Toxicology, Faculty of Veterinary Medicine, Zagazig University, Alzeraa Street Postal Code 44511, Zagazig City, Sharkia Province, Egypt

**Keywords:** antioxidant enzymes, fish, phagocytosis, Roundup, Stomp

## Abstract

**Aim:**

The current study was directed to investigate the immunotoxic and oxidative stress effects of Roundup and Stomp herbicides and their combination on Nile catfish (*Clarias gariepinus*).

**Materials and Methods:**

The experiment was carried out on 120 fish that randomly divided into four equal groups with three replicates: The first group kept as control, the second group exposed to 1/2 96 h lethal concentration 50 (LC_50_) of Roundup, the third group exposed to 1/2 96 h LC_50_ of Stomp, and the fourth one exposed to a combination of Roundup and Stomp at previously-mentioned doses. The experiment was terminated after 15 days; blood samples were obtained at 1^st^, 8^th^, and 15^th^ days of treatment where the sera were separated for estimation of antioxidant enzymes. Meanwhile, at 15^th^ day of exposure part of blood was collected from all groups with an anticoagulant for evaluation of phagocytic activity, then the fish were sacrificed, and specimens from the liver of all groups were obtained for histopathological examination.

**Results:**

Our results indicated that both herbicides either individually or in combination elucidated significant decrease in phagocytic activity that was highly marked in group exposed to both herbicides. Furthermore, our data elicited an obvious elevation in the levels of superoxide dismutase (SOD), catalase (CAT), and glutathione peroxidase (GPx). Meanwhile, the data depicted reduction in levels of reduced glutathione (GSH) and glutathione-S-transferase (GST). Histopathological investigation of liver proved the aforementioned results.

**Conclusion:**

It could be concluded that either Roundup or Stomp alone cause significant deleterious effects on aquatic vertebrates. However, the use of their combination enhanced their toxic effects. Toxicity can end up in humans through the food chain.

## Introduction

Worldwide, pesticide use has increased dramatically over the past two decades [1]. Pesticides have become some of the most frequently occurring organic pollutants of agricultural soils, ground and surface water, causing ecological imbalances [2] that may have toxicological effects on natural ecosystems, especially aquatic systems [3]. They cause damage to non-target organisms including fish [4]. The runoff of pesticides (insecticides, herbicides, and fungicides) from agricultural lands is a key concern for the health of aquatic organisms [5].

Herbicides are actively used in terrestrial and aquatic ecosystems to control unwanted weeds, and their use has generated serious concerns about the potential adverse effects of these chemicals on the environment and human health [6]. They are the most widely used chemicals in agriculture as they are account for about 40% of the pesticides volume used worldwide. Roundup is one of the most common herbicides used in agriculture and also used in forestry and horticulture including home use [7]. Moreover, it is being the most popular and widely used herbicide in most parts of the world [8]. The indiscriminate use of Roundup associated with careless handling, accidental spillage, or discharge of untreated effluents into natural waterways has caused harmful effects on aquatic life and may have contributed to long-term biological effects [9].

Stomp is liquid emulsive herbicide of the dinitroaniline type; its active ingredient is pendimethalin [10]. Pendimethalin is highly toxic to fish and aquatic invertebrates [11].

Reactive oxygen species (ROS) generation is continuously produced in biological systems either as side products of aerobic metabolism or products of specialized systems designed to produce ROS [12]. Perez *et al*. [13] examined the effects of three widely used pesticides that have been widely detected in aquatic systems, in which they found that binary mixtures elicited synergistic responses; however, aquatic organisms are rarely exposed to only one single contaminant but typically to mixtures of numerous pesticides with varying constituents in varying concentrations and concentration ratios. Biochemical markers, such as lipid peroxidation (LPO) and antioxidant enzymes, are widely used to assess the toxic stress, integrity of the immune system, and tissue damage in different organisms [14].

Although there is a considerable amount of information available on toxicity of individual herbicides to fish and aquatic invertebrates, there is less information on toxicity of herbicide mixtures to these organisms. Furthermore, the impacts of Stomp in fish have so far undergone little research, and there is still a great need to properly assess the impact of Stomp on fish. Therefore, the present work was conducted to shed some light on the hazardous effects of Roundup- and Stomp-based herbicides and their combination on Nile catfish (*Clarias gariepinus*).

## Materials and Methods

### Ethical approval

All animal-related procedures were carried out in accordance with the Ethical Committee of Zagazig University.

### Fish and experimental design

A total number of 120 Nile catfish (*C. gariepinus*) with a body weight ranged from 90 to 115 g were used. Fish were obtained from the ponds of the Central Laboratory of Aquaculture Research, Abbassa, Abou-Hammad, Sharkia, Egypt. Fish were apparently healthy and free from skin lesions or external parasites; they were maintained in glass aquaria (50x40x150 cm capacity) having 180 L of dechlorinated tap water. Each aquarium provided with aerator, thermostatically controlled with heater and thermometer. Fish were acclimatized for 2 weeks to the laboratory environment. Fish were fed 3 times daily on a basal diet contained 35.4% crude protein (from Hendrix Co.) The amount of food per day was 3% of fish body weight. The experiment was conducted for 2 weeks.

The fish were divided into four equal groups: The first group kept as control, the second group exposed to 1/2 96 h lethal concentration 50 (LC_50_) of Roundup (14.5 mg/L) [15], whereas the third one exposed to 1/2 96 h LC_50_ of Stomp (420 µg/L) [16]. The fourth group exposed to a combination of 1/2 96 h LC_50_ of both of Roundup and Stomp.

### Tested compounds

#### Roundup (glyphosate-based herbicide)

It was obtained in a commercial form Roundup. It is containing 48% emulsion concentration (EC), obtained from Monsanto Agriculture Company, USA.

Empirical Formula: C_3_H_8_NO_5_P

Chemical Formula: N- (phosphonomethyl) glycine.

State: Clear, viscous amber-colored solution.

#### Stomp (pendimethalin-based herbicide)

Stomp^®^, it is containing 50% EC (BASF PLC), an orange-yellow liquid emulsive herbicide of the dinitroaniline type [17]. It contains the inert components (50%), as petroleum solvents (naphthalene and ethylene dichloride).

Empirical formula: C_13_H_19_N_3_O.

Chemical formula: [n-(1-ethylpropyl)-3,4dimethyl-2,6-dinitrobenzenamine].

State: Orange-yellow solution.

### Sampling and measurements

Blood samples were obtained at 1^st^, 8^th^, and 15^th^ days of treatment; blood samples were collected from the caudal blood vessel [18] using sterile syringes, which was left to be clotted at room temperature followed by centrifugation at 3000 rpm, for 15 min for serum separation for biochemical studies, another part of blood at 15^th^ day of exposure was collected in glass tubes containing 10% ethylenediaminetetraacetic acid (EDTA) solution and centrifuged at 3000 rpm for 30 min. The plasma was removed completely followed by separation of leukocytes for evaluation of phagocytic activity, and then fish were sacrificed by decapitation and dissected. Specimens from livers of the treated and control groups were obtained and preserved in 10% neutral-buffered formalin for histopathological examination.

### Immunotoxic study

#### Chemicals used for studying the phagocytic activity

1. Microbial strain: *Candida albicans* was kindly supplied by the Department of Bacteriology, Mycology and Immunity, Faculty of Veterinary Medicine, Zagazig University. It was used in a concentration of 5 × 10^6^ CFU/ml.
2. Culture media:
  - Sabouraud’s dextrose agar
    Media used for isolation and cultivation of *C. albicans*. This media was prepared according to the method described by Cruickshank *et al*. [19]. The ingredients were dissolved in distilled water at 121°C for 15 min. after being autoclaved and cooled to 45°C, chloramphenicol was added at concentration of 100 mg/L to the medium, pH was adjusted to be around 5.
  - Roswell Park Memorial Institute 1640 medium (RPMI 1640) with l-glutamine
    The medium was obtained from GIBCO limited, UK, in a sterile patent preparation. The pH was adjusted to 7.2 by adding 2 g/L sodium bicarbonate. This medium was used for leukocytes cultivation.
3. Buffers
  - Phosphate-buffered saline (PBS)
    PBS was prepared according to the method described by Cruickshank *et al*. [19]. The PBS pH was adjusted to 7.0 and the sterilized by autoclaving at 121°C for 15 min. This buffer was used for anticoagulant preparation, cell washing, and for serial dilution in killing assay.
  - Anticoagulant buffer
    EDTA solution (10%) in distilled water; it was used as 1:5 volumes of blood.

### Evaluation of phagocytic activity by Wilkinson [20], Lucy and Lary [21]

1. Preparation of Hank’s balanced salt solution (HBSS) [19].
2. Preparation of *C. albicans* for determining the phagocytic activity
  *C. albicans* was grown on Sabouraud’s 2% dextrose broth for 48 h at 37°C to obtain the organism in the yeast phase only. The cultures were spinned at 1500 rpm for 10 min and the deposit washed twice with PBS, and filtered through sterile gauze. The yeasts were resuspended in HBSS so as to give a concentration of 5 × 10^6^ CFU/ml.
  The yeasts were killed by heating at 100°C in water bath for 30 min. A large batch was prepared and divided into small aliquots sufficient for each test. These were stored at −20°C until needed.
3. Preparation of leukocytes suspension for phagocytosis assay [20,21].
  - Peripheral blood leukocytes suspension was prepared by sterile procedures
  - 2.5 ml of blood were collected after 15 days of exposure and carefully layered on surface of Ficoll hypaque solution 1.077 density gradient equal volumes in sterile plastic tube
  - Centrifugation at 2400 rpm for 30 min at 18-20°C. The mononuclear cells form a white opaque band at Ficoll plasma interface. This layer was aspirated by sterile Pasteur pipette and placed in sterile plastic tube containing HBSS
  - The separated cells washed 3 times in HBSS at 2500, 1500, and 1000 rpm, respectively, each for 10 min
  - Sedimented cells were suspended in 1 ml of the RPMI media containing 1% fetal calf serum.
4. Preparation for phagocytosis assay
  In sterile plastic tubes, put 0.25 ml leukocyte suspension, 0.25 ml heat-killed *C. albicans*, 0.25 ml pooled serum, and 0.25 ml HBSS.
  The tubes were incubated at 37°C for 30 min. They were centrifuged at 2500 rpm for 5 min, and the supernatant was removed with Pasteur pipette leaving a drop into which the sediment was resuspended. Smears were prepared from the deposit, dried in air, and stained with Leishman’s stain.

### Evaluation of phagocytic activity

Under a light microscope using oil immersion lens, 10 fields, each containing about 20 phagocytes, were examined. The total number of phagocytic cells and the number of phagocytes with ingested yeast cells in individual phagocytes were determined to calculate the percentage of phagocytosis and the phagocytic index (PI). The mean particle number associated with each cell represents the PI.


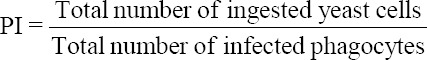


The number of cells ingesting *Candida* represents the phagocytic percentage (P%).


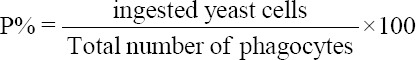


### Biochemical studies

Determination of superoxide dismutase (SOD) activity according to the method described by Misra and Fridivich [22] and modified by Packer and Glazer [23], catalase (CAT) activity according to the method described by Sinha [24], reduced glutathione (GSH) according to the method described by Beutler [25] and modified by Beutler *et al*. [26], glutathione-S-transferase activity (GST) according to Habig *et al*. [27], and glutathine peroxidase activity (GPx) according to Puertas *et al*. [28].

### Histopathological study

Histopathological examination of livers of control and treated groups that were collected and fixed in 10% buffered neutral formalin solution, dehydrated in gradual ethanol (70-100%), cleared in xylene, and embedded in paraffin. 5 µ thick paraffin sections were prepared, and then routinely stained with hematoxylin and eosin dyes according to Suvarna *et al*. [29], and then examined microscopically.

### Statistical analysis

The obtained data were analyzed using the SPSS software, version 16 (SPSS Inc., Chicago, IL, USA). Groups’ data were compared by one-way analysis of variance. The statistical significance was accepted at (p<0.05).

## Results

### Effect of Roundup and Stomp on phagocytic activity

**Figure-1:**
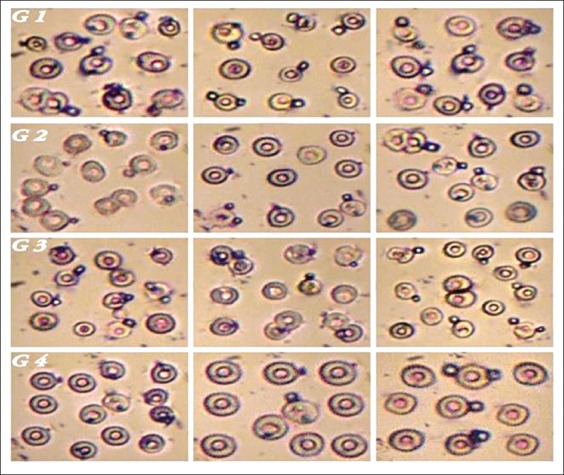
Photograph of phagocyte cells showing the effect of Roundup, Stomp, and both on phagocytic activity of *Clarias gariepinus* after 15 days of exposure. (G1) Phagocyte cells engulfing yeast cells in *C. gariepinus* of control group. (G2) Phagocyte cells showing destruction and inhibition of phagocytosis in group exposed to Roundup. (G3) Phagocyte cells showing destruction and inhibition of phagocytosis in group exposed to Stomp. (G4) Phagocyte cells showing severe destruction and inhibition of phagocytosis in group exposed to combination of Roundup and Stomp.

The data illustrated in Table-1 and Figure-1 indicated the effect of Roundup and/or Stomp treatment on phagocytic activity of *C. gariepinus* leukocytes against yeast cells at 15^th^ day of exposure. The phagocytic percent (P%) was significantly reduced in the Roundup-treated group compared to the control one, while there was a non-significant decrease of the phagocytic percent in group treated with Stomp when compared with the control group. On the other hand, the phagocytic percent in group treated with both Roundup and Stomp showed an obvious significant decrease comparing with control group. In addition, the data illustrated clear significance difference between all treated groups when compared with each other. Regarding the PI, there was a significant decrease in all treated groups when compared to the control one.

**Table-1:** Changes in phagocytic percent (p%) and PI in *C. gariepinus* exposed to Roundup, Stomp, and both after 15 days of exposure (mean±SE) (n=30).

Group	Treatment	Phagocytic percent (p%)	PI
1	Control	33.46±3.51^a^	1.35±0.05^a^
2	1/2 LC_50_ Roundup	20.16±1.03^b^	0.964±0.03^b^
3	1/2 LC_50_ Stomp	27.08±1.53^a^	1.01±0.03^b^
4	1/2 LC_50_ Roundup and 1/2 LC_50_ Stomp	13.5±1.55^c^	0.936±0.06^b^

Means within the same column having the different superscripts are significantly different at (p<0.05). PI=Phagocytic index, SE=Standard error, LC_50_=Lethal concentration 50

### Effect of Roundup and Stomp on SOD and CAT activities

Concerning the SOD activity in serum of *C. gariepinus*, our study showed a significant increase in fish exposed to the combination of both Roundup and Stomp at 1^st^ day of exposure. While there was no significant increase in fish exposed to Roundup or Stomp separately compared with the control group. At 8^th^ day of exposure, SOD activity increased significantly in fish exposed to Roundup, but there was no significant increase in the Stomp-treated group, while the increase in SOD activity was highly obvious in the combined treated group. Furthermore, the three treated groups depicted significance difference when compared with each other. Meanwhile, at 15^th^ day of exposure, the SOD activity elevated significantly in all treated groups compared to the control one (Table-2).

**Table-2:** Changes in SOD and CAT activities of *C. gariepinus* exposed to Roundup, Stomp, and both at 1^st^, 8^th^, and 15^th^ days of exposure (mean±SE) (n=30).

Parameters	SOD (unit/mg protein)			CAT (µmol H_2_O_2_ decomposed/ml)		
	
	1^st^	8^th^	15^th^	1^st^	8^th^	15^th^
Control	40.26±1.16^a^	42.50±0.78^a^	44.35±2.85^a^	28.38±2.75^a^	31.89±0.55^a^	44.63±1.20^a^
1/2 LC_50_ Roundup	47.89±3.03^a^	52.60±1.65^b^	51.57±1.54^b^	67.37±3.77^c^	49.16±3.60^b^	48.16±1.8^ab^
1/2 LC_50_ Stomp	46.28±1.29^ab^	43.74±3.60^a^	51.22±1.82^b^	47.03±0.31^b^	44.71±1.83^b^	46.91±2.6^ab^
1/2 LC_50_ Roundup and 1/2 LC_50_ Stomp	52.03±3.60^b^	60.44±0.25^c^	56.52±2.33^b^	69.50±1.46^c^	56.26±1.00^c^	51.98±1.3^b^

Means within the same column having the different superscripts were significantly different (p<0.05). SOD=Superoxide dismutase, CAT=Catalase, *C. gariepinus=Clarias gariepinus,* SE=Standard error, LC_50_=Lethal concentration 50

Regarding the CAT activity, the present study showed a significant elevation in fish exposed to Stomp while the elevation was obviously significant in groups exposed to Roundup and the combination of both herbicides comparing with the control group at 1^st^ day of exposure.

At 8^th^ day of exposure, fish exposed to Roundup or Stomp separately showed a significant increase in CAT activity, while the elevation was highly obvious in groups exposed to the combination of both herbicides comparing with the control group and also when comparing with Roundup and Stomp-treated groups. At 15^th^ day of exposure, the elevation in CAT activity was significant only in fish exposed to the combination of Roundup and Stomp, but there was no significant increase at other treated groups comparing with the control group (Table-2).

### Effect of Roundup and Stomp on the activities of reduced GSH, GST, and GPx

Regarding the GSH level in serum of *C. gariepinus*, the current study demonstrated a significant decrease in Roundup exposed group and obviously significant decrease in the combined treated group at 1^st^ day of exposure compared to the control group, and also, there was significance difference in GSH level when comparing the treated groups with each other. While at 8^th^ and 15^th^ day of exposure the decrease in GSH level was significant in all treated groups when compared to the control one (Table-3).

**Table-3:** Changes in GSH, GST, and GPx activities of *C. gariepinus* exposed to Roundup, Stomp, and both at 1^st^, 8^th^, and 15^th^ days of exposure (Mean±SE) (n=30).

Parameters	GSH (mg/ml)			GST (U/l)			GPx (mU/ml)		
		
	1^st^	8^th^	15^th^	1^st^	8^th^	15^th^	1^st^	8^th^	15^th^
Control	4.73±0.05^a^	4.44±0.25^a^	4.53±0.38^a^	0.39±0.017^a^	0.46±0.036^a^	0.45±0.02^a^	19.42±0.73^c^	13.65±0.89^c^	11.39±0.53^c^
1/2 LC_50_ Roundup	3.28±0.50^b^	2.75±0.33^b^	2.83±0.24^b^	0.31±0.012^a^	0.35±0.03^a^	0.27±0.06^b^	23.19±0.53^b^	21.28±0.64^b^	21.89±0.86^b^
1/2 LC_50_ Stomp	4.39±0.31^a^	2.84±0.30^b^	2.88±0.24^b^	0.35±0.08^a^	0.38±0.03^a^	0.38±0.02^a^	20.97±0.49^bc^	20.80±0.87^b^	13.44±1.07^c^
1/2 LC_50_ Roundup and 1/2 LC_50_ Stomp	1.43±0.19^c^	2.06±0.36^b^	2.53±0.28^b^	0.30±0.017^a^	0.22±0.04^b^	0.14±0.03^c^	28.34±1.52^a^	24.32±0.65^a^	28.23±2.55^a^

Means within the same column having the different superscripts were significantly different (p<0.05). LC_50_=Lethal concentration 50, GSH=Reduced glutathione, GST=Glutathione-S-transferase activity, GPx=Glutathione peroxidase, *C. gariepinus=Clarias gariepinus*, SE=Standard error

Concerning the GST activity, no significant variations versus control were observed in all treated groups at 1^st^ day of exposure. At 8^th^ day of exposure, fish exposed to the combination of both Roundup and Stomp showed a significant reduction in GST activity, but there was no significant alteration in other treated groups comparing with control one. While at 15^th^ day of exposure, the reduction in GST activity was obvious in Roundup-treated group and highly obvious in group treated with the combination of both herbicides, besides, our data revealed that there was significance difference in GST level when comparing the three treated groups with each other, but no significant reduction was observed in Stomp-treated group compared to the control one (Table-3).

Regarding the GPx activity, fish exposed to Roundup showed a significant increase at all durations of exposure compared to the control one. In Stomp-treated group, the GPx activity showed a significant elevation at the 8^th^ day of exposure but did not show any significant elevation at other durations of exposure when compared with the control group. Fish exposed to the combination of both herbicides showed an obviously significant elevation compared to the control group in all durations of exposure (Table-3).

### Histopathological findings

**Figure-2:**
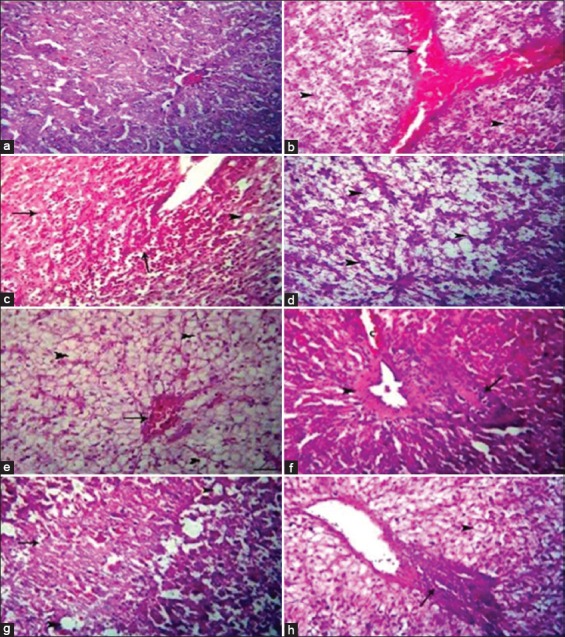
Liver from different groups: Control shows normal hepatocytes and sinusoidal architectures (a), Roundup shows severe congestion in hepatoportal blood vessels (arrow) (b), area of necrosis invaded with numerous leukocytes and erythrocytes (arrows) (c), and macrovesicular steatosis (d). Stomp shows congestion and severe hydropic degeneration and vacuolation in the hepatocytes (arrowheads) (e) and portal area with periductal fibrosis and round cells infiltration (arrow), and hyalinization in the wall of blood vessel (arrowhead) (f). Both Roundup and Stomp shows extensive coagulative necrosis (arrow) and perivascular edema (g) and aggregation of round cells in the portal area (arrow) (h). Hematoxylin and eosin × Scale bar = 25 µm.

The liver of the control revealed normal hepatocytes and sinusoidal architectures (Figure-2a). However, the liver of Roundup showed severe congestion in hepatoportal blood vessels (Figure-2b). Multifocal areas of coagulative necrosis invaded with numerous leukocytes and erythrocytes were visualized (Figure-2c). Severe hydropic degeneration and macrovesicular steatosis were detected (Figure-2d). The liver of Stomp showed moderate congestion, severe hydropic degeneration, and vacuolation in the hepatocytes (Figure-2e). The portal areas revealed periductal fibrosis and round cells infiltration besides hyalinization in the wall of blood vessels (Figure-2f). The reported lesions in both exposures to Roundup and Stomp were severe and represented by extensive coagulative necrosis and perivascular edema (Figure-2g). Intense interstitial and portal aggregations of round cells were noticed (Figure-2h).

## Discussion

Aquatic environments are commonly impacted by various pesticides (including herbicides, fungicides, and insecticides) from different sources. Fish species are described as suitable monitors for the effects of noxious compounds because of their ecological and economical relevance [9]. In addition, changes at cellular and biochemical levels are among the most sensitive biological responses reported after fish exposure to aquatic pollutants [30].

The immune system of vertebrates, including that of fish, reacts with a particular sensitivity to xenobiotic exposure [31]. Phagocytic activity is a primitive defense mechanism and an important component of the non-specific immune system [32]. Phagocytosis is a response helpful to assess the immunological impact of environmental pollutants [33]. The obtained results revealed that the phagocytic activity was decreased significantly in Roundup and/or Stomp-treated fish. Our results concurred with those obtained by Kreutz *et al*. [34] in silver catfish exposed to 10% of the 96 h-LC_50_ glyphosate. A similar result was also observed in rainbow trout exposed to pendimethalin [35]. This decrease in phagocytic activity may be attributed to high soluble concentrations of herbicide disturb cell phagocytic activity. In fact, the membrane integrity of cells could be impaired by uptake of chemical compounds in organisms, which could interfere with the fluidity of cell membranes restricting the deformation of the membrane essential to the phagocytic endocytosis process [36] On the contrary of our result, Mohamed [37] studied the effect of the herbicide Roundup on *Biomphalaria alexandrina* hemocytes and revealed a highly significant increase in the phagocytic activity that may lead to cytotoxic effects.

Matricon-Gondran and Letocart [38] speculated that these cytotoxic effects may be due to the changes in the sensitivity of intercellular adhesion molecules involved in the ingestion of foreign materials, resulting in increase of phagocytic activity.

Herbicides have the potential to introduce ROSs into biological systems, leading to oxidative stress on non-target organisms [39]. Fish, as many other vertebrates, are endowed with defensive mechanisms to counteract the harmful effects of ROS resulting from the metabolism of various chemicals or xenobiotics [40].

ROSs are naturally produced during several cellular pathways of the aerobic metabolism, including oxidative phosphorylation, electron transport chains in mitochondria and microsomes, the activity of oxidoreductase enzymes, which produce ROS as intermediates or final products, and even immunological reactions, such as active phagocytosis [41].

SOD and CAT enzymes have related functions [42]. SOD is a group of metalloenzymes that plays a crucial antioxidant role and constitutes the primary defense against the toxic effects of superoxide radicals in aerobic organisms. SOD catalyzes the transformation of superoxide radicals to hydrogen peroxide and water and is the first enzyme to cope with oxyradicals [43]. CAT is an enzyme that is located in the peroxisomes and facilitates the removal of hydrogen peroxide, which is metabolized to molecular oxygen and water [44]. Nwani *et al*. [45] assessed the alteration in LPO and antioxidant enzymes activities. They found that induction of oxidative stress in the blood and gill cells were evidenced by increased LPO level while antioxidants, namely, SOD and CAT responded in a concentration-dependent manner.

In the present work, the SOD and CAT activities increased after exposure of *C. gariepinus* to Roundup and/or Stomp. The increase in these enzyme activities is most likely a response to the increased ROS generation induced by pesticide toxicity [46]. This hypothesis was corroborated by Monteiro *et al*. [42], who described the simultaneous induction of SOD and CAT activities in *Brycon cephalus* exposed to methyl parathion and Pieniazek *et al*. [47], who found an increment in CAT levels in human erythrocytes after exposure to glyphosate and Roundup. The increases in the serum SOD and CAT activities that were observed in this study may be meant to neutralize the overproduction of superoxide anions and hydrogen peroxides due to the oxidative stress induced by herbicides. Our findings supported by a previous observation of Noori [48], who mentioned that the failure in the neutralization events of oxidative status results in oxidative stress which leads to the cell death by LPO.

Reduced GSH is a main non-protein thiol and is a primary reductant in cells [49]. The main role of GSH is based on its ability to protect cells against oxidative damage caused by free radicals by providing a reduced medium to cells; GSH also serves as an important substrate for the reductive detoxification of reactive intermediates such as hydrogen peroxide or hydroperoxides. Consequently, estimation of the level of this compound should provide essential data describing the processes which happen in cells [50]. In the current study, the GSH level was significantly decreased in the Roundup and/or Stomp exposed fish. This result was in accordance with those obtained by Danion *et al*. [51] in *Oncorhynchus mykiss* exposed to pendimethalin and also with previous studies, which reported a GSH depletion with other pesticides and herbicides in different cellular populations *in vitro* [50,52] and *in vivo* [53] but were opposed to others [54,55]. The decrease in GSH content may be related to utilization of this antioxidant in the metabolism of herbicides through GSH-Px activity. The action of this enzyme was strictly linked with the GSH concentration because it catalyzed the reaction between GSH and hydrogen peroxide, resulting in the formation of GSH disulfide. Thus, it could be assumed that herbicides, by lowering the level of GSH and decreasing the GSSG-Red activity, led to an oxidative imbalance and induced oxidative processes.

GSTs are a group of enzymes that catalyze the conjugation of reduced GSH with a variety of electrophilic metabolites and are involved in the detoxification of both reactive intermediates and oxygen radicals [44]. It has been demonstrated that the activity of these enzymes may be enhanced in the liver of fish exposed to a variety of pollutants, and even low-level organic contamination can lead to increased hepatic GST activity in fish [56]. However, in the present work, *C. gariepinus* exposed to the herbicides Roundup and Stomp showed decrease in GST activity.

This decrease may reinforce the idea of the presence of oxidants that would lead to the inactivation of the enzymatic activity [57] considering that GST is sensitive to products of the Haber–Weiss reaction [58]. The inhibition of GST was also observed in the liver of goldfish after 96 h exposure to Roundup [6]. Conversely, when *Prochilodus lineatus* was exposed to Roundup [59] found a significant increase in liver GST after 24 and 96 h exposure.

GPx catalyzes the reduction of hydrogen peroxide and lipid peroxides and is considered an efficient protective enzyme against LPO at the expense of GSH [60]. In this work, our result revealed a significant elevation in GPX activity in serum of *C. gariepinus* exposed to Roundup and/or Stomp. This increase is in consistence with that result obtained by Gehin *et al*. [61] in HaCaT cultured cells exposed to glyphosate or Roundup. This increase may indicate that the antioxidant pathway was stimulated, probably due to the increased production of peroxides. Although this enzyme acts principally in the removal of organic peroxides, it is also involved in the metabolization of hydrogen peroxide [62]. The aforementioned results of our study concerning immunotoxicity and oxidative stress came in harmony with histopathological changes in the liver after exposure to Roundup, which revealed severe congestion in hepatoportal blood vessels, multifocal areas of coagulative necrosis invaded with numerous leukocytes and erythrocytes and severe hydropic degeneration and macrovesicular steatosis were detected. This result was nearly similar to that found in *Piaractus mesopotamicus* [63] and *P. lineatus* [64]. In this work, the most frequent encountered types of degenerative changes are those of hydropic degeneration, cloudy swelling, vacuolization, and focal necrosis. The liver of the exposed fish had slightly vacuolated cells showing evidence of fatty degeneration. Necrosis of some portions of the liver tissue that were observed probably resulted from the excessive work required by the fish to get rid of the toxicant from its body during the process of detoxification by the liver. The inability of fish to regenerate new liver cells may also have led to necrosis [65].

While the liver of *C. gariepinus* exposed to Stomp showed moderate congestion, severe hydropic degeneration, and vacuolation in the hepatocytes. The portal areas revealed periductal fibrosis and round cells infiltration besides hyalinization in the wall of blood vessels. This result parallel to findings of Nabela *et al*. [66], who reported that these changes may be attributed by the enterohepatic pathway of the Stomp (pendimethalin-based herbicide).

## Conclusion

The current study implicated that using Roundup and Stomp separately caused significant deleterious effects on aquatic vertebrates. However, the use of their combination exaggerated their obvious toxic effects. In turn, their toxicity can end up in humans through the food chain. The suitable controlled and regular use of herbicides is recommended, to obtain the beneficial effects of these resources without polluting the environment and without leaving their residues in food and water sources with potentially negative effects on human health.

## Authors’ Contributions

GGM, FES, and AHAH generated the concept and designed the study. GGM and WME carried out the practical part and drafted the manuscript. GGM revised and approved the final manuscript.
